# An Exfoliated Graphite-Based Electrochemical Immunosensor on a Dendrimer/Carbon Nanodot Platform for the Detection of Carcinoembryonic Antigen Cancer Biomarker

**DOI:** 10.3390/bios9010039

**Published:** 2019-03-08

**Authors:** Azeez O. Idris, Nonhlangabezo Mabuba, Omotayo A. Arotiba

**Affiliations:** 1 Department of Applied Chemistry, University of Johannesburg, Doornfontein 2028, Johannesburg, South Africa; idrisalone4real@gmail.com (A.O.I.); nmabuba@uj.ac.za (N.M.); 2 Centre for Nanomaterials Science Research, University of Johannesburg, Doornfontein 2028, Johannesburg, South Africa

**Keywords:** carcinoembryonic antigen, immunosensor, cancer, polypropylene imine, exfoliated graphite electrode

## Abstract

An electrochemical immunosensor for the quantification of carcinoembryonic antigen (CEA) using a nanocomposite of polypropylene imine dendrimer (PPI) and carbon nanodots (CNDTs) on an exfoliated graphite electrode (EG) is reported. The carbon nanodots were prepared by pyrolysis of oats. The nanocomposites (PPI and CNDTs) were characterized using X-ray powder diffraction (XRD), Raman spectroscopy, Fourier transform infrared spectroscopy (FTIR), high-resolution transmission electron microscopy (HRTEM) and scanning electron microscopy (SEM). The proposed immunosensor was prepared on an exfoliated graphite electrode sequentially by drop coating CNDTs, the electrodeposition of G2-PPI (generation 2 poly (propylene imine) dendrimer), the immobilization of anti-CEA on the modified electrode for 80 min at 35 °C, and dropping of bovine serum albumin (BSA) to minimize non-specific binding sites. Cyclic voltammetry was used to characterize each stage of the fabrication of the immunosensor. The proposed immunosensor detected CEA within a concentration range of 0.005 to 300 ng/mL with a detection limit of 0.00145 ng/mL by using differential pulse voltammetry (DPV). The immunosensor displayed good stability and was also selective in the presence of some interference species such as ascorbic acid, glucose, alpha-fetoprotein, prostate-specific antigen and human immunoglobulin. Furthermore, the fabricated immunosensor was applied in the quantification of CEA in a human serum sample, indicating its potential for real sample analysis.

## 1. Introduction

The fabrication of an effective and efficient diagnostic tool for the detection of cancer and monitoring its progression during therapy is a vital task in biomedical analysis. The possibilities of such analysis in oncology have been enhanced by the discovery of tumor biomarkers such as alpha-fetoprotein, CA125, prostate-specific antigen, cytokeratin 19 fragment and carcinoembryonic antigen (CEA) to name a few [1,2].

CEA is a tumor marker associated with liver, ovarian, breast, colorectal and lung cancer [3]. The average concentration of CEA in a healthy human is 5 µg/L and a concentration above 20 µg/L is an indication of cancer [4]. Changes in CEA concentrations in a patient with colorectal cancer can be used to monitor different stages of the disease and to detect early recurrence after surgery [3,4]. It is important to highlight that among all the existing methods for the quantification of CEA, immunoassay is used as a benchmark, or standard, for single-protein detection because of the reactivity of an antibody with its corresponding antigen to form an immunocomplex reaction [5]. Such methods include chemiluminescence immunoassay [6], surface-enhanced Raman scattering immunoassay [7] and electrochemical immunoassay [8,9]. Among these methods, the electrochemical immunosensor has been of interest to scientists because of its fascinating properties, which include high sensitivity, fast response, low cost, short diagnostic time and miniaturization [3,10].

The crucial steps in the construction of an electrochemical immunosensor are the immobilization of the immunoreagent on the electrode surface and the enhancement of the electrochemical signal produced by the immunoconjugates. To enhance electrochemical signals, smart nanomaterials are used as a stage for the immobilization of antibodies or antigens for the construction of immunosensor for cancer biomarkers or biosensors [2,11,12,13]. Different analogues of nanomaterials, such as nanowires, nanoflakes, nanotubes, nanofibers, quantum dots, dendrimers, and nanodots, have helped to improve the surface area and to facilitate electron transport on the surface of an electrode. For instance, Gu et al. reported the use of gold nanoparticles with a ferrocene derivative for the construction of an electrochemical sensor for carcinoembryonic antigens; the sensor displayed good performance and stability for more than four weeks [1]. Similarly, gold nanoparticles, in conjunction with silver nanoparticles, were employed in the construction of a sandwich-type immunosensor. It was reported that the Ag/Au nanostructure displayed good electrochemical activity in the working potential range, which enhanced the current response and a detection limit of 8 pgm/L [9]. In another report, a silver nanocluster alongside a horseradish peroxidase nanocomposite was used in the construction of an immunosensor for the detection of CEA; the developed nanocluster/enzyme signal probe was successfully used for the detection of CEA with a limit of detection of 0.5 pg/mL [8].

Carbon nanodots (CNDTs) are quasi-spherical analogues of carbon material with a particle size of less than 10 nm. It consists of an amorphous or nanocrystalline core with predominantly sp^2^ hybridization and an oxidized carbon surface containing different functional groups such as carboxyl, hydroxyl and aldehyde groups [14,15]. Carbon nanodots are an important member in the family of carbon nanomaterials because of its interesting properties, such as low toxicity, high aqueous solubility, photophysical properties, low cost, robust chemical inertness, ease of synthesis, and excellent biocompatibility [15,16,17]. These unique properties of CNDTs attracted its application in different fields such as bio-imaging [18], chemical sensing and biosensing [19,20,21], drug delivery [22], dye sensitizers [23], catalysis [24] and fuel cells [25].

Few reports can be found in the literature on the application of CNDTs in the construction of biosensors. For instance, Zhang et al. reported the application of dendrimer functionalized carbon nanodots for the electrochemical detection of alpha-fetoprotein [26]. In another report, horseradish peroxidase was immobilized on carbon nanodot-/CoFe-layered double hydroxides and employed for the electrochemical quantification of hydrogen peroxide [27]. Furthermore, thionine and chitosan-entrapped carbon nanodot films were used for the fabrication of an electrochemical biosensor in wastewater toxicity assessments [28]. More so, carbon nanodots have been employed as a peroxidase nanozyme for biosensing [29]. The remarkable results obtained from these reports spurred us towards employing carbon nanodots for this study.

Furthermore, another class of nanomaterial that has fascinating properties is poly (propylene imine) dendrimer (PPI). It is a polymer with a tree-like morphological structure consisting of high-density terminal groups and a peripheral functionality [30,31,32]. The properties of dendrimer include chemical stability, self-assembly, poly-valency, low cytotoxicity, host–guest supramolecular features, solubility, high surface area, exterior functional groups and excellent biocompatibility features [30,31,32]. These unique properties lend dendrimers to be used for various applications such as bio-imaging and gene delivery [33], as catalysts [34], in nanomedicine [35], in biomedicine [36], as a water treatment membrane [37] and as a DNA biosensor [38]. Moreover, we have explored the application of dendrimers as a smart nanomaterial for the detection of various health-related diseases such as HIV, cholera, urea and alpha-fetoprotein [39,40,41,42].

In this novel study, we report the concept of a synergic combination between polypropylene imine dendrimers and carbon nanodots in the construction of an immunosensor for the quantification of carcinoembryonic antigens. More so, the host–guest supramolecular properties and biocompatibility properties of dendrimers are employed to facilitate the immobilization of the antibody. We anticipated that this approach to CEA quantification would improve immunosensor performance. To the best of our knowledge, no report has been documented for the electrochemical quantification of CEA using CNDTs/PPI modified on an exfoliated graphite electrode.

## 2. Experimental

### 2.1. Materials and Instruments

Natural graphite (NG) flakes, generation 2 (G2) PPI dendrimer, carcinoembryonic antigen (CEA), anti-carcinoembryonic antigen, prostate-specific antigen, urea, human immunoglobulin, bovine serum albumin, ascorbic acid, Na_2_HPO_4_, NaH_2_PO_4_, HAuCl_4_, KCl and HNO_3_ were purchased from Sigma Aldrich (Johannesburg, South Africa). Alpha-fetoprotein was purchased from Celtic Diagnostic (Cape Town, South Africa). The following analytical techniques were used for characterization: X-ray diffractometry (XRD) on a Rigaku Smartlab X-ray diffractometer (Wilmington, MA, USA), and Raman spectrum was carried out on a Raman microscope (PerkinElmer Raman micro 200, Waltham, MA, USA) with ×50 objective. The SEM and TEM electron micrographs were taken using (TESCAN, Vega 3 XMU, Czech Republic and JEOL 2100 HRTEM 200 V, Tokyo, Japan). Fourier transform infrared spectroscopy (FTIR) was done on PerkinElmer Spectrum 100 spectrometer (Waltham, MA, USA). Ivium Compactstat potentiostat (Eindhoven, The Netherlands) was used for all electrochemical measurements.

### 2.2. Fabrication Procedure for the Exfoliated Graphite Electrode

NG flakes (approximately 300 µm particle size) were intercalated with a mixture of concentrated nitric acid and sulfuric acid in a volume ratio of 1:3 for 24 h at room temperature to form a graphite-intercalated compound (GIC) [43]. The intercalated material was washed several times with deionized water until a pH of 6.5 was attained; after this, it was subjected to thermal shock at 800 °C for half a minute so as to force the intercalated material out of the graphite lattice, thus rupturing the layers. This process formed a puffed carbon material called exfoliated graphite (EG). The prepared EG was used to form a pellet by compressing 0.4 g of it at a pressure of 70 kPa for 4 h. The exfoliated graphite electrode was fabricated from the compressed pellets using copper wire, a glass rod and conductive silver paint. The procedure for the fabrication of the EG electrode was done by using a puncher to cut the EG pellet into a circle 3 mm in diameter. After this, the top layer of the copper wire was scraped off with a blade to remove any oxide on the surface, while the other end was coiled to form a circular surface. A conductive silver paint was rubbed on to the circular coil so that the 3 mm diameter of the exfoliated graphite pellet could sit on it. It was then left to dry at room temperature After drying, the electrode was inserted into a glass tube and further coated with an Araldite epoxy resin (an insulator), leaving only the basal plane of the pellet. The EG electrode was polished using P1500-grit emery sheets to obtain a smooth uniform surface.

### 2.3. Synthesis of the Carbon Nanodot

The carbon nanodot was synthesized by weighing 10 g of oats, which was further crushed and pyrolyzed in a muffle furnace at 400 °C for 2 h [14]. The color of the oats changed from white to black after 2 h. It was allowed to cool at room temperature and finally crushed to a fine powder. The product obtained was dispersed in ultrapure water and centrifuged several times at 7800 revolutions per minute (rpm) to remove the larger particles. The carbon nanodot aqueous suspension was filtered and dried in an oven for 24 h at 80 °C.

### 2.4. Preparation of the Immunosensor

**Scheme 1:**
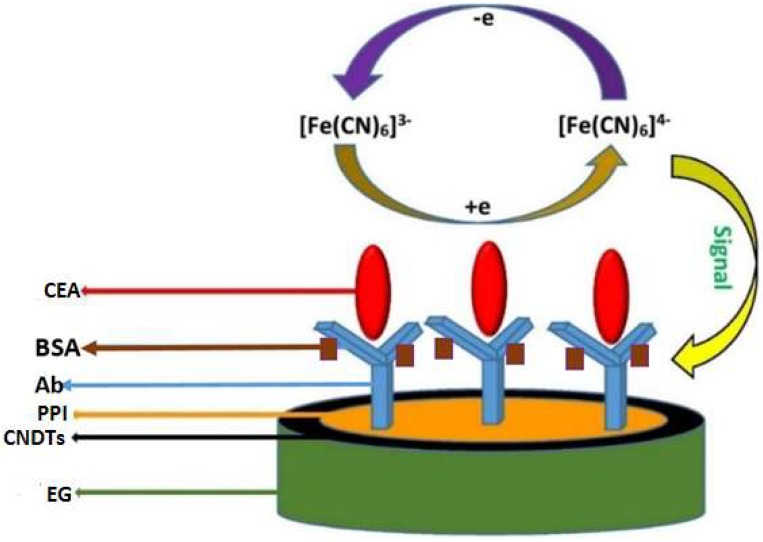
Processes involved in the preparation of the immunosensor.

The fabricated exfoliated graphite electrode (EG) was polished using emery sheets to obtain a smooth, uniform surface. A 30 mg mass of the synthesized carbon nanodots was carefully weighed and gently dispersed in 5 mL of dimethylformamide, and it was ultrasonicated for 60 min. Hereafter, 20 µL of the dispersed carbon nanodot solution was drop-dried on the fabricated exfoliated graphite electrode at room temperature; the electrode was assigned as EG-CNDTs. Furthermore, G2-PPI was electrochemically deposited onto the EG-CNDTs from an electrolytic solution of 5 mM G2-PPI by running a potential from −400 to 1100 mV for ten cycles at a scan rate of 50 mV/s [42]. This electrode was labelled as EG/CNDTs@PPI. Thereafter, 20 µL of aqueous glutaraldehyde (25%) was drop-dried on EG/CNDTs@PPI as a cross-link for the immobilization of anti-CEA. Furthermore, 20 µL of 300 ng/mL anti-CEA solution, prepared in a phosphate buffer solution (PBS) of pH 7.2, was immobilized on EG/CNDTs@PPI. The electrode was ascribed as EG-CNDTs/anti-CEA and was then incubated with 0.25% of bovine serum albumin (BSA) for 3 h. The constructed immunosensor was labelled as EG/CNDTs@PPI/anti-CEA/BSA and stored at 4 °C when not in use. The schematic for the construction of the immunosensor is presented in Scheme 1.

### 2.5. Experimental Measurements

Electrochemical measurements were carried out on a three-electrode system configured of a working electrode (3 mm exfoliated graphite electrode), reference electrode (Ag/AgCl (3 M KCl)), and a counter electrode (platinum wire). The experimental parameters for the cyclic voltammetry (CV) measurements were an E-step of 10 mV, current range of 1 µA, potential from −0.6 V to 1.2 V, and a scan rate of 50 mV/s in a solution of 1 mM [Fe(CN)_6_]^3−/4−^ prepared in 0.1 M KCl as the supporting electrolyte. The experimental parameters for differential pulse voltammetry (DPV) were a pulse time of 10 ms, a pulse amplitude of 10 mV, E-step of 10 mV, current range of 1 µA, a scan rate of 50 mV/s, and equilibration time of 5 s.

## 3. Results and Discussion

### 3.1. Characterization of the Nanomaterials

**Figure 1:**
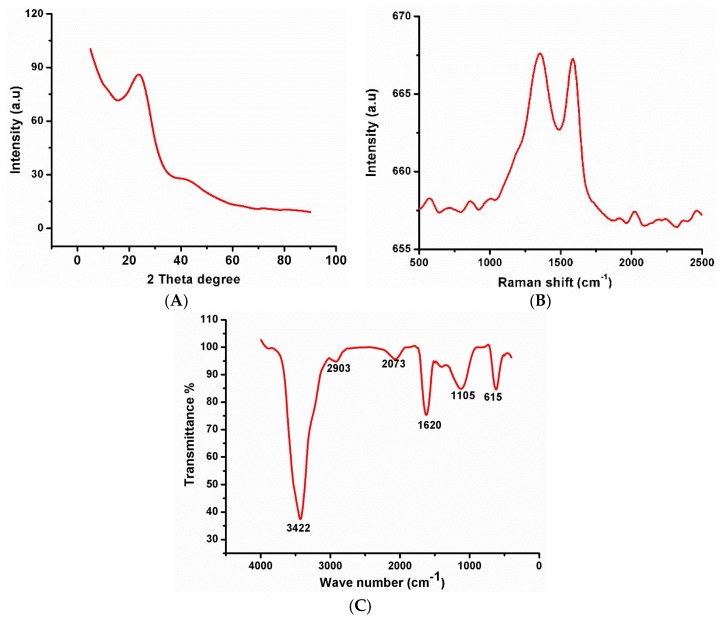

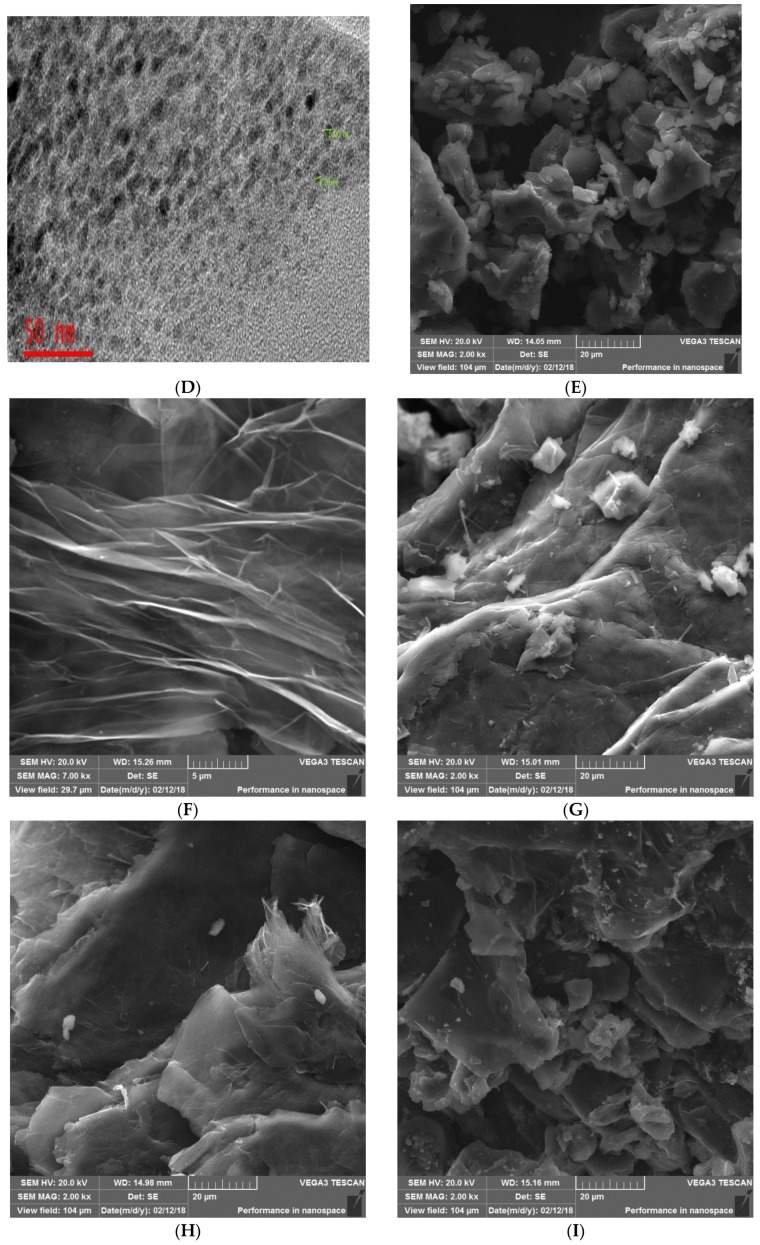
(**A**) XRD spectrum of carbon nanodots (CNDTs). (**B**) Raman spectrum of CNDTs. (**C**) Fourier transform infrared spectroscopy (FTIR) of CNDTs. (**D**) TEM image of CNDTs. (**E**) SEM image of CNDTs. (**F**) SEM image of the exfoliated graphite electrode. (**G**) SEM image of CNDTs deposited on exfoliated graphite electrode. (**H**) SEM image of G2-PPI electrodeposited on the exfoliated graphite electrode. (**I**) SEM image of CNDTs@G2-PPI on the exfoliated graphite electrode.

The XRD pattern of the synthesized CNDTs is depicted in Figure 1A. Two diffraction peaks were observed at 2θ values of 24.63° and 42.64°, the former represented (111) lattice plane and the latter represented a diamond phase in the CNDTs [44]. The Raman spectrum of the synthesized CNDTs in Figure 1B shows two broad peaks at 1355 and 1591 cm^−1^, which corresponded to the D and G bands of the graphite. The ratio of D to G peaks, which is a measure of disorder in the crystal structure, was calculated to be 0.85. This ratio confirmed a high degree of disorder in the CNDTs. The functional groups present in the synthesized CNDTs were investigated using FTIR, as depicted in Figure 1B. The peak at 3430 cm^−1^ was attributed to the –OH vibration of water, the peaks at 2923 and 2073 cm^−1^ corresponded to an aliphatic (C-H) stretch band and (-C≡N), indicating the presence of amino-containing functional groups. Moreover, the three absorption peaks at 1620, 1105, and 615 cm^−1^ were attributed to the carbonyl (C=O) stretching vibration, symmetric carboxylate stretch, and aryl group (=C-H) of the CNDTs, respectively [45,46].

An oval/spherical shape structure of CNDT was observed from the TEM image (Figure 1D). Similarly, an aggregated structure of CNDT was revealed by the SEM image depicted in Figure 1E. The morphology of the exfoliated graphite electrode before and after the modification process was investigated using scanning electron microscopy (SEM). The SEM micrograph of the compressed exfoliated graphite was flower-like in shape, as shown in Figure 1F. After modifying the compressed EG with CNDTs, aggregates of CNDTs were dispersed on the compressed EG, as shown in Figure 1G. Similarly, electrodeposition of G2-PPI on compressed EG revealed the spherical shape of the dendrimer, as depicted in Figure 1H. The spherical and oval-like shape of CNDTs and G2-PPI were evenly dispersed on the compressed EG, as presented in Figure 1I.

### 3.2. Electrochemical Characterization and Optimization

**Figure 2:**
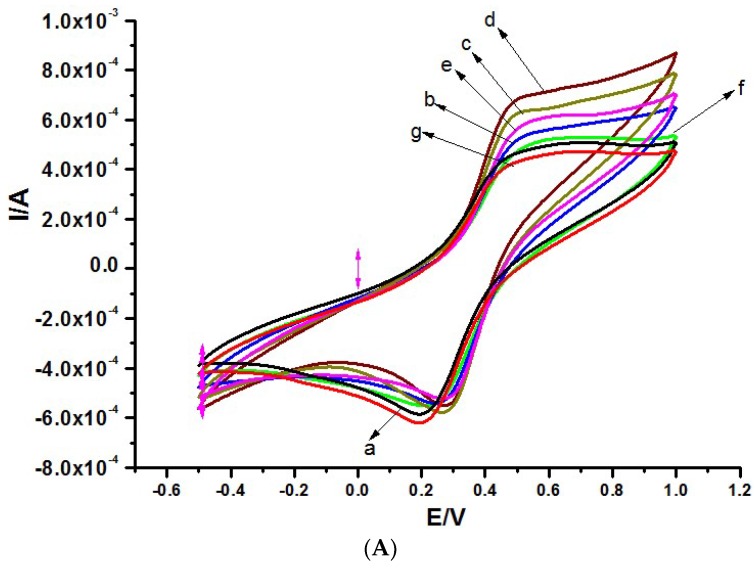

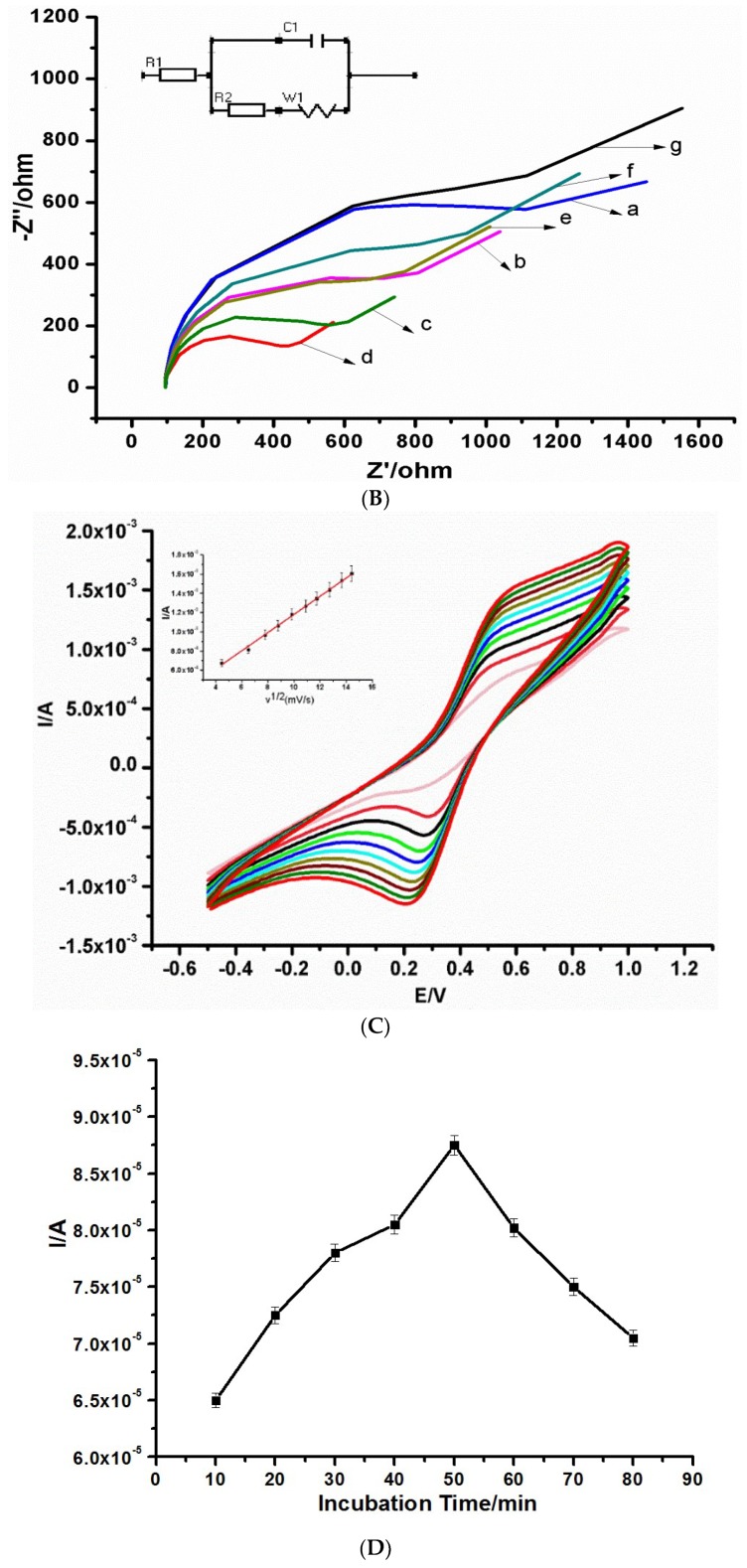

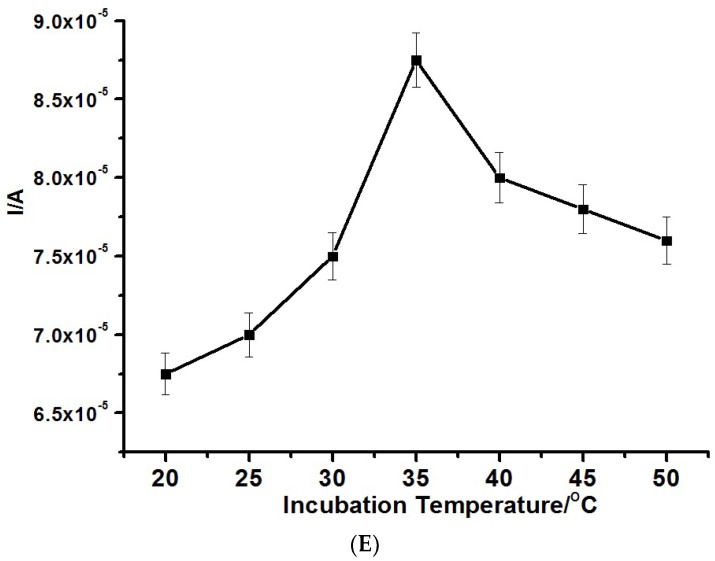
(**A**) CV and (**B**) EIS of (**a**) Bare exfoliated graphite electrode (EG), (**b**) EG/PPI, (**c**) EG/CNDTs, (**d**) EG/CNDTs@PPI, (**e**) EG/CNDTs@PPI/Anti-CEA, (**f**) EG/CNDTs@PPI/Anti-CEA/BSA, and (**g**) EG/CNDTs@PPI/Anti-CEA/BSA/CEA. (**C**) CV of different scan rates of immunosensor (EG/CNDTs@PPI/Anti-CEA/BSA) in solution containing 1 mM [Fe(CN)_6_]^3−/4−^ prepared in 0.1 M KCl. The inset is a plot of current versus the square root of scan rates. (**D**) Optimization of CEA detection incubation time. (**E**) Optimization of CEA detection incubation temperature in solution containing 1 mM [Fe(CN)_6_]^3^^−/4−^ prepared in 0.1 M KCl.

The fabrication steps of the immunosensor were interrogated using cyclic voltammetry in a solution containing 1 mM [Fe(CN)_6_]^3−/4−^ prepared in 0.1 M KCl, as shown in Figure 2A. The bare exfoliated graphite gave a reversible redox peak (Figure 2Aa). About a 24% enhancement in current signal was noticed after modifying the exfoliated graphite electrode with G2-PPI; this electrode was labelled EG-PPI (Figure 2Ab). The increase in peak current signal was due to an enhancement in the surface area of the electrode and an improvement in the conductivity of the exfoliated graphite electrode. On modifying exfoliated graphite with CNDTs, a 48% current enhancement was observed, owing to the accelerating electron transfer kinetics of the CNDTs on bare exfoliated graphite; this electrode was ascribed as EG-CNDTs (Figure 2Ac). A synergic increase in peak currents of 71% (relative to the bare electrode) was obtained after modification with the nanocomposite of CNDTs@PPI (Figure 2Ad). This can be from the interplay of surface area, conductivity, and acceleration of electron transport on the bare exfoliated graphite electrode. The electrode was tagged EG/CNDTs@PPI. As expected, there was a decrease in peak current signal by 14.2% after the immobilization of 300 ng/mL anti-CEA on EG/CNDTs@PPI, as shown in (Figure 2Ae). This was due to the non-conducting property of anti-CEA, which retarded the flow of electrons at the electrode interface, and this electrode was branded as EG/CNDTs@PPI/anti-CEA. A similar event unfolded on incubating 0.25% BSA on EG/CNDTs@PPI/anti-CEA to block the non-specific binding, a reduction in peak current signal of 16.6% was obtained. This electrode was marked as EG/CNDTs@PPI/anti-CEA/BSA and was referred to as the immunosensor. After the immunosensor was incubated in 100 ng/ml of CEA, the peak current, as expected, decreased (Figure 2Ag) owing to the immunocomplex formed at the electrode surface, indicating a successful binding event.

Table 1 shows the electrochemical impedance spectroscopy (EIS) data of the immunosensor preparation as fitted using the Randle–Sevcik model. The EIS results presented in Figure 2B and Table 1 agreed with the CV (Figure 2A). It is important to highlight that an increase in current connotes lower charge transfer resistance or less resistance to current flow. The lowest charge transfer resistance was obtained from the nanocomposite of CNDTs@PPI (Figure 2Bd), which was in concord with the highest peak current obtained (from Figure 2Ad).

The stability of the platform—EG/CNDTs@PPI/Anti-CEA/BSA—employed in the construction of the immunosensor was interrogated as depicted in Figure 2C. It was observed that the peak currents and the square roots of scan rates were in direct proportionality with a correlation coefficient, R^2^ = 0.9961. This proportionality predominately indicated a diffusion-controlled system, which is thus suitable for electroanalysis.

The incubation time and temperature of the immunosensor were optimized. Incubation time depended predominantly on the kinetic features of the immunochemical reaction and mass transfer of immunoreagents. The fabricated immunosensor was incubated with 200 ng/mL CEA using differential pulse voltammetry, the peak current increased progressively from 10 to 50 min, as depicted in Figure 2D. A reduction in peak current after this time was an indicator that binding was completed. Thus, 50 min was chosen as the optimum incubation time.

Figure 2E shows the effect of temperature (from 15 to 50 °C) on the incubation process. The peak current increased proportionately with an increase in incubation time from 15 to 35 °C, and the peak current of 35 °C was chosen as the working temperature. The decrease in current after this temperature may be a result of the denaturing of the protein (antibody).

### 3.3. Analytical Application of the Immunosensor

**Figure 3:**
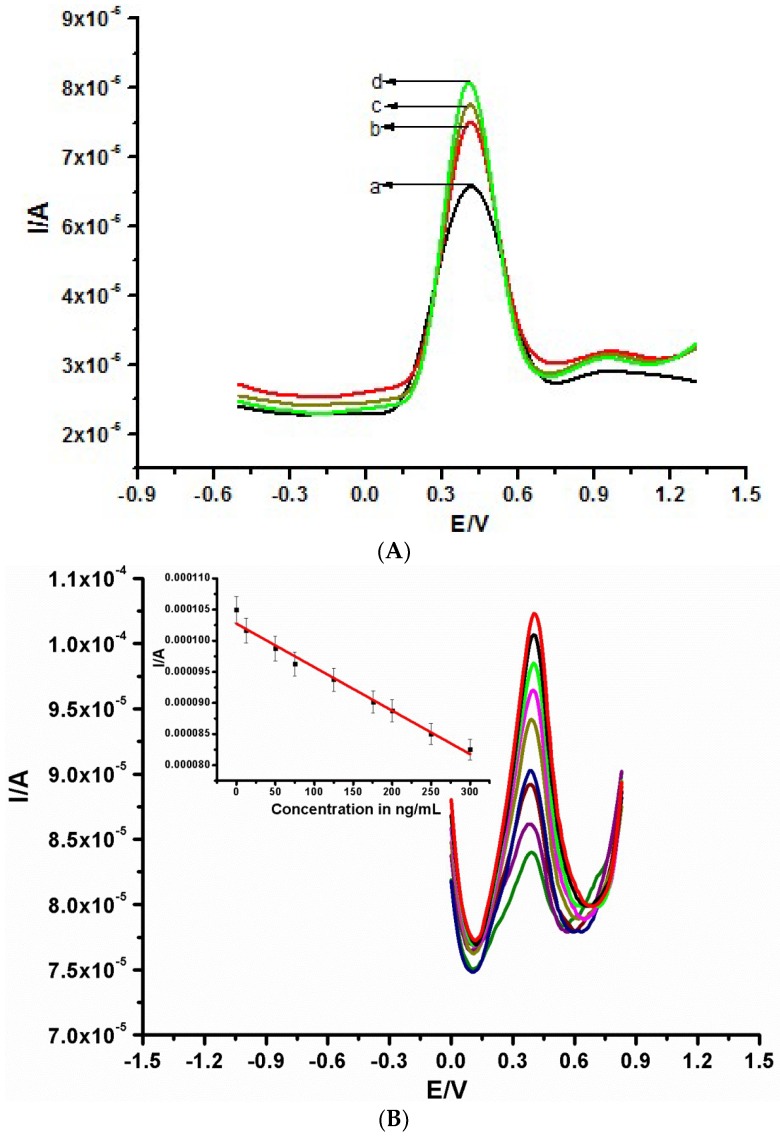
(**A**) Differential pulse voltammetry (DPV) of various constructed immunosensors for the detection of 300 ng/mL Carcinoembryonic antigen (CEA) fabricated from (**a**) EG/Antibody@BSA, (**b**) EG/PPI@Antibody/BSA, (**c**) EG/CNDTs@Antibody/BSA, and (**d**) EG/CNDTs@PPI@Antibody/BSA. (**B**) DPV of various concentrations of CEA from 0.005 to 300 ng/mL in 1 mM [Fe(CN)_6_]^3−/4−^.

The immunosensor was prepared on various platforms as controls for the determination of 50 ng/mL CEA (Figure 3A). The maximum peak current signal was obtained from the nanocomposite from CNDTs@PPI in relation to other platforms, strengthening the synergetic effect and optimum performance of the CNDT and PPI platforms.

The immunosensor was utilized to quantify different concentrations of CEA under the optimized experimental conditions. An inverse proportionality between the CEA concentration and current was noticed with differential pulse voltammetry (Figure 3B). This was due to the fact that an increase in the amount of bound CEA led to a more constrained electron flow that resulted from the non-conducting properties of the CEA or the immunocomplex. The formula used in calculating the limit of detection was $\frac{3\times SD}{S}$, where SD and *S* represent the standard deviation of the blank and the slope of the calibration graph, respectively. The following were derived from the calibration: a linear concentration range of 0.005 to 300 ng/mL with a linear regression equation of Y(µA) = 102.7 − 0.070*x* (DPV), a detection limit of 0.00145 ng/mL (±4.67 × 10^−6^) (DPV), and a correlation coefficient of 0.9834.

The low detection limit of the fabricated label-free immunosensor can be attributed to the following: the possible supramolecular chemistry (electrostatic attraction and host–guest chemistry) between the dendrimer and the antigen; the characteristic of CNDT as nanozyme (nanomaterials with enzyme-like characteristics) [29]; the biocompatibility of both CNDTs and PPI; and the synergic features of CNDTs@PPI. The bio-recognition efficiency of bioreceptors depended on its molecular conformation or integrity. That is, the way the molecules are conformed in nature. This conformation may be affected by covalent bonds, which can deactivate a functional group or make rigid certain parts of the molecules, thus, reducing binding and folding. Since supramolecular interactions protect the natural conformity of molecules, it is envisaged that a biosensor development that allows more supramolecular interactions (like the dendrimer-antibody in this report) enables a closer representation of nature (biomimicry) and, thus, better bio-recognition.

Table 2 shows a comparison between the immunosensor in this study with others reported in the literature for CEA. Although the synergy of gold and platinum biosensors gave a better detection limit than the reported immunosensor, this report had a lower cost compared to the expensive gold and platinum. However, the proposed immunosensor compares well (or performs better in some cases) with other reports because of the unique analytical merits of the nanomaterials (CNDTs@PPI), which includes: (i) The CNDT was prepared from a natural oat, which is also an inexpensive carbon source (green method); (ii) the electrode used was fabricated from cheap carbon material (natural graphite); (iii) there was a wide linear range of 0.005 to 300 ng/mL; (iv) the immunosensor involved a simple two-step preparation method; (v) electrostatic attraction between the positive and negative charge of the two modifiers assisted in obtaining a lower detection limit; (vi) the modifiers employed have biocompatibility properties; and (vii) the electrode employed for this study was cheaper in comparison to glassy carbon electrodes that are commonly used.

### 3.4. Stability, Selectivity, and Repeatability of the Immunosensor

**Figure 4:**
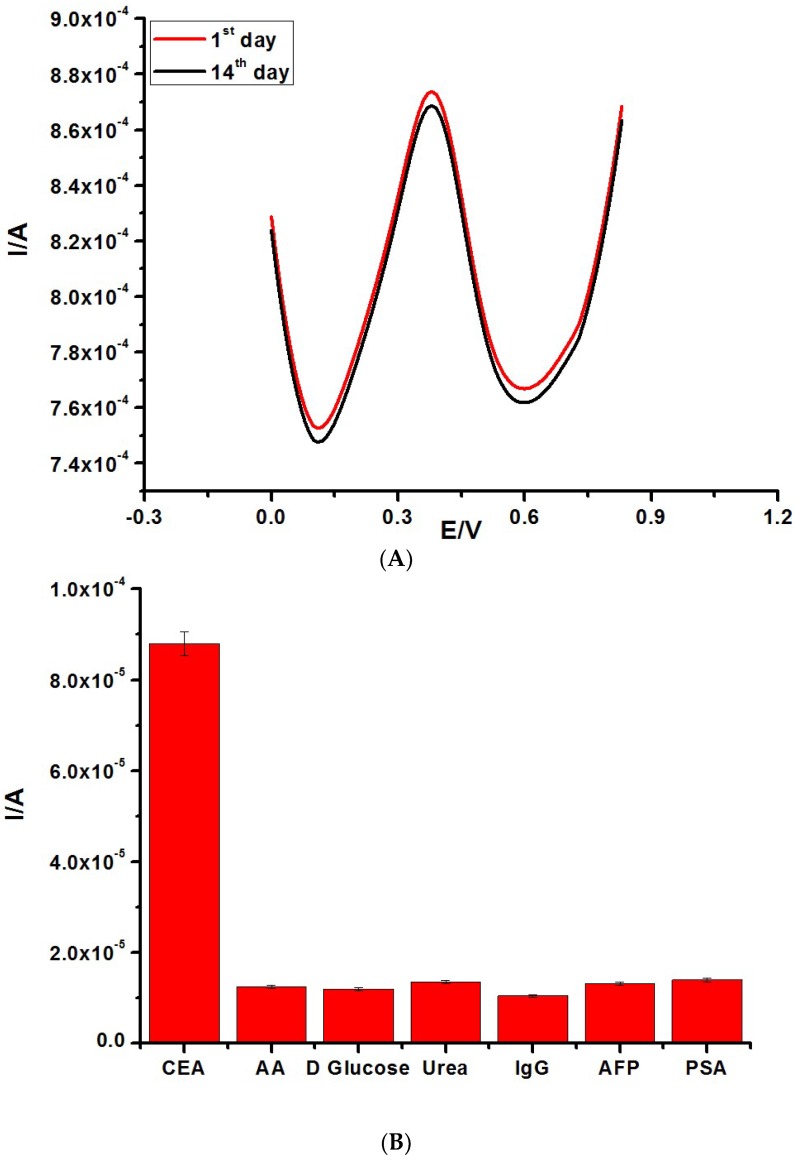

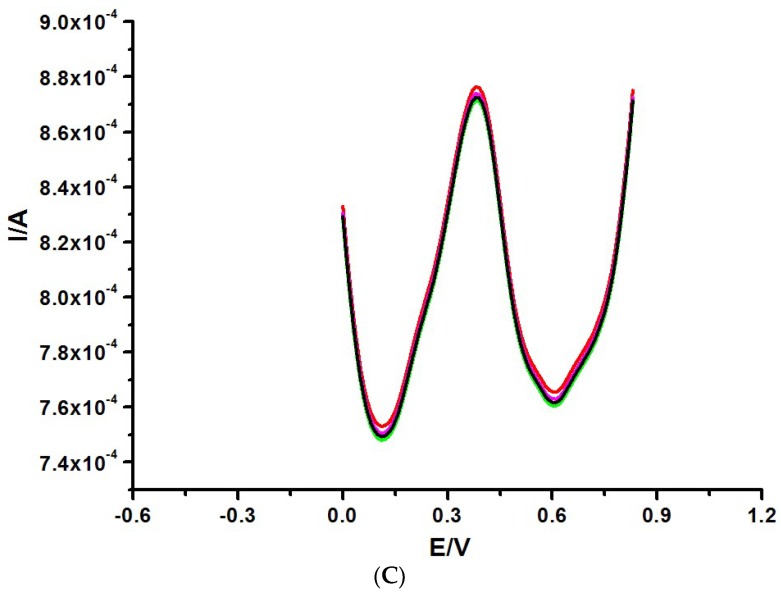
(**A**) Stability test of the fabricated immunosensor from the 1st to the 14th day. (**B**) Selectivity result of the constructed immunosensor in solution containing 1 mM [Fe(CN)_6_]^3−/4−^. (**C**) DPV of different immunosensors in 1 mM [Fe(CN)_6_]^3−/4−^ prepared in 0.1 M KCl.

The stability of the constructed label-free electrochemical immunosensor was interrogated after storage for two weeks at 4 °C. There was less than a 4% decrease in electrochemical signal for the detection of the same concentration of CEA (200 ng/mL) after two weeks of storage, as shown in Figure 4A. The selectivity of the immunosensor was carried out in 200 ng/mL CEA solution and in the presence of a 400 ng/mL concentration of possible interfering species, such as prostate-specific antigen, alpha-fetoprotein, human immunoglobulin G, ascorbic acid (AA), BSA, and urea (Figure 4B). The biosensor exhibited a reasonable selectivity, owing to a current variation of less than 11% observed for all interferents.

The repeatability of the immunosensor was carried out by constructing four immunosensors from various electrodes, and each fabricated electrochemical sensor was used for the detection of 200 ng/mL of CEA (Figure 4C). A low relative standard deviation (RSD) of less than 4% denoted that the method proposed can be repeated and, thus, it is of analytical significance.

### 3.5. Application of the Immunosensor

The immunosensor was applied for the detection of CEA in human serum (×100 dilution in phosphate buffer solution). CEA concentrations of 0.5, 50, and 100 ng/mL were added to the diluted human serum samples, and the analysis of each sample was carried out 5 times. At a 95% confidence limit, the percentage recovery and RSD were 99.96%–100.03% and 0.0192%–0.1313%, respectively (Table 3). The results revealed that the proposed label-free immunosensor had analytical merit or viability in the determination of CEA in the serum sample.

## 4. Conclusions

In this work, a novel combination of carbon nanodots and poly(propyleneimine) dendrimers has been employed as an electrode material for the fabrication of a label-free cancer biomarker immunosensor for carcinoembryonic antigens. The nanocomposite platform assisted in obtaining a low detection limit, a good reproducibility, and a good selectivity. This work reveals the applicability of these nanocomposites in biosensor design. The biocompatibility properties of the two modifiers assisted in the detection of CEA in the serum sample. The proposed immunosensor is anticipated to assist in the quantification of different cancer biomarkers and other electrochemical applications.

## Figures and Tables

**Table 1:** The result obtained from the circuit fitting for the construction of the immunosensor depicted in Figure 2B.

Circuit Element	EG	EG/CNDTs	EG/PPI	EG/CNDTs@PPI	EG/CNDTs@PPI Antibody	EG/CNDTs@PPI Antibody	EG/CNDTs@PPI Antibody + CEA
*R_s_* (Ω	95.5	92.7	93.1	90.2	91.8	92.7	89.5
*R_ct_* (Ω)	964.8	385.8	523.0	293.9	519.5	658.4	972.6

**Table 2:** Comparing the performance of the reported immunosensor to previous reports in the literature.

Immunosensor Fabrication	Linear Range (ng/mL)	Detection Limit (ng/mL)	References
AuNPs	1–200	0.5	[47]
AuNPs-HRP	0.5–7	0.01	[48]
Polyaniline-AuNPs	0.1–1000	0.007	[49]
Au/Pt	0.01–200	0.00011	[50]
NH_2_-G/Thi/AuNPs	0.02–80	0.008	[9]
AuNPs/FCN	0.05–20	0.01	[1]
CNDTs/PPI	0.005–300	0.00145	Present work

NH_2_-G/Thi/AuNPs-amino functionalized graphene (NH_2_-G)/thionine (Thi)/gold nanoparticles(AuNPs), and AuNPs/FCN—gold nanoparticles ferrocene derivatives.

**Table 3:** Application of immunosensors in human serum samples.

Serum Sample (ng/mL)	The Addition Content (ng/mL)	The Detection Content (ng/mL)	RSD (%, n = 6)	Recovery (%)	Confidence Intervals
0.00	5.0	5.01,5.00,4.99, 5.02, 4.97	0.0192	99.96	4.98–5.01
	50.0	49.94, 49.97, 50.02, 50.06, 50.03	0.0482	100.01	49.95–50.04
	100.0	100.06, 99.97, 100.03, 100.08, 99.99	0.1313	100.03	99.93–100.06
